# Familial Experience With Hirschsprung's Disease Improves the Patient's Ability to Cope

**DOI:** 10.3389/fped.2022.820976

**Published:** 2022-03-07

**Authors:** Sanne J. Verkuijl, Rob J. Meinds, Alida F. W. van der Steeg, Cornelius E. J. Sloots, Ernst van Heurn, Ivo de Blaauw, Wim G. van Gemert, Marieke J. Witvliet, Karin M. Vermeulen, Monika Trzpis, Paul M. A. Broens

**Affiliations:** ^1^Division of Pediatric Surgery, Department of Surgery, University Medical Center Groningen, University of Groningen, Groningen, Netherlands; ^2^Anorectal Physiology Laboratory, Department of Surgery, University Medical Center Groningen, University of Groningen, Groningen, Netherlands; ^3^Department of Gastroenterology and Hepatology, Medisch Spectrum Twente, Enschede, Netherlands; ^4^Princess Maxima Center for Pediatric Oncology, Utrecht, Netherlands; ^5^Department of Pediatric Surgery, Erasmus Medical Centre, Sophia Children's Hospital, Rotterdam, Netherlands; ^6^Department of Pediatric Surgery, Emma Children's Hospital, Academic Medical Centre and VU University Medical Centre, Amsterdam, Netherlands; ^7^Division of Pediatric Surgery, Department of Surgery, Radboudumc, Amalia Children's Hospital, Nijmegen, Netherlands; ^8^Department of Pediatric Surgery, University Medical Centre Maastricht, University of Maastricht, Maastricht, Netherlands; ^9^Department of Pediatric Surgery, Wilhelmina Children's Hospital, University Medical Centre Utrecht, Utrecht, Netherlands; ^10^Department of Epidemiology, University Medical Center Groningen, University of Groningen, Groningen, Netherlands

**Keywords:** Hirschsprung, quality of life, psychosocial development, inheritance, coping

## Abstract

**Introduction:**

Familial occurrence of Hirschsprung's disease may have a positive effect on patients' ability to cope with the disease. The aim was to compare long-term bowel function and generic quality of life between patients with familial and non-familial Hirschsprung's disease.

**Methods:**

This was a nationwide, cross-sectional study in which we included all 830 Hirschsprung patients of 8 years and older who had undergone surgery between 1957 and 2015. We excluded patients with a permanent stoma, intellectual disability, or an unknown or foreign address. We requested patients to complete the validated pediatric or adult Defecation and Fecal Continence questionnaire and the Child Health Questionnaire Child Form-87, or the World Health Organization Quality of Life-100 Assessment Instrument.

**Results:**

We analyzed 336 Hirschsprung patients, 15.8% of whom were familial cases and 84.2% were non-familial cases. After adjusting for aganglionic length, sex, and age, patients with familial Hirschsprung's disease were twice more likely to suffer from constipation (OR = 2.47, 95% CI, 1.21–5.05, *p* = 0.013). The quality of life of the pediatric patients was comparable, but in adult patients the energy/fatigue, thinking/learning/concentration, and work capacity facets showed better scores in the familial patients with Hirschsprung's disease of the rectosigmoid (*p* = 0.029, *p* = 0.024, *p* = 0.036, respectively).

**Conclusions:**

Different facets of generic quality of life are better in adult patients with familial Hirschsprung's disease of the rectosigmoid. It seems that familial experience with the disease influences patients' coping abilities positively.

## Introduction

Hirschsprung's disease is a congenital bowel disorder characterized by a lack of ganglion cells in the distal bowel (1). The aganglionic bowel usually leads to bowel obstruction and requires surgery at an early age. It is known that about 20% of the cases of Hirschsprung's disease are familial, with an overall familial recurrence risk between 4 and 8% (2–4). Furthermore, it is known that familial Hirschsprung's disease is associated with longer lengths of the aganglionic segment (2–4).

The genetics of Hirschsprung's disease have received considerable attention in the past years. Nevertheless, we still know little about the differences in bowel function between patients with and without familial Hirschsprung's disease, except for rare reports on progressive disease in siblings (4). Since modern surgery has led to substantial improvement in the survival of Hirschsprung patients, the responsibilities of pediatric physicians and surgeons regarding postoperative function and quality of life has increased accordingly (5). One can imagine that it might be easier to talk about bowel function problems, like constipation and fecal incontinence, with relatives who suffer from Hirschsprung's disease themselves. It has also been suggested that familial coping strategies have a positive influence on patients' own ability to cope (6, 7). Thus, one may expect that familial occurrence of Hirschsprung's disease may improve patients' ability to cope.

We therefore hypothesize that, differences in aganglionic length apart, the quality of life of patients with familial Hirschsprung's disease is better than that of non-familial patients. The aim of this study was to compare long-term bowel function and generic quality of life between patients with familial and non-familial Hirschsprung's disease.

## Materials and Methods

### Study Design

This was a nationwide, cross-sectional study on 830 patients who had undergone surgical resection for Hirschsprung's disease in one of the six Dutch pediatric surgical centers between 1957 and 2015. We only included patients who were older than seven years at the time the study commenced. We excluded patients without a known Dutch postal address, with a permanent stoma, patients who were intellectually disabled, or deceased patients. This was based on information from the medical records or by self-report of the patients. After the patients had returned the informed consent form, we sent them two questionnaires. We asked the parents or caretakers of patients younger than 18 years to complete the questionnaire together with their children. One investigator searched the medical files for perioperative clinical data. Analyses of the data generated in the study were published previously (8, 9). For the purpose of the current analysis, we excluded 10 patients with an ultrashort aganglionic segment on account of the small number of familial and non-familial patients in this subgroup. Our study population consisted of Hirschsprung patients with aganglionosis of the rectosigmoid, long-segment, or total colon.

### Questionnaires

**Figure 1:**
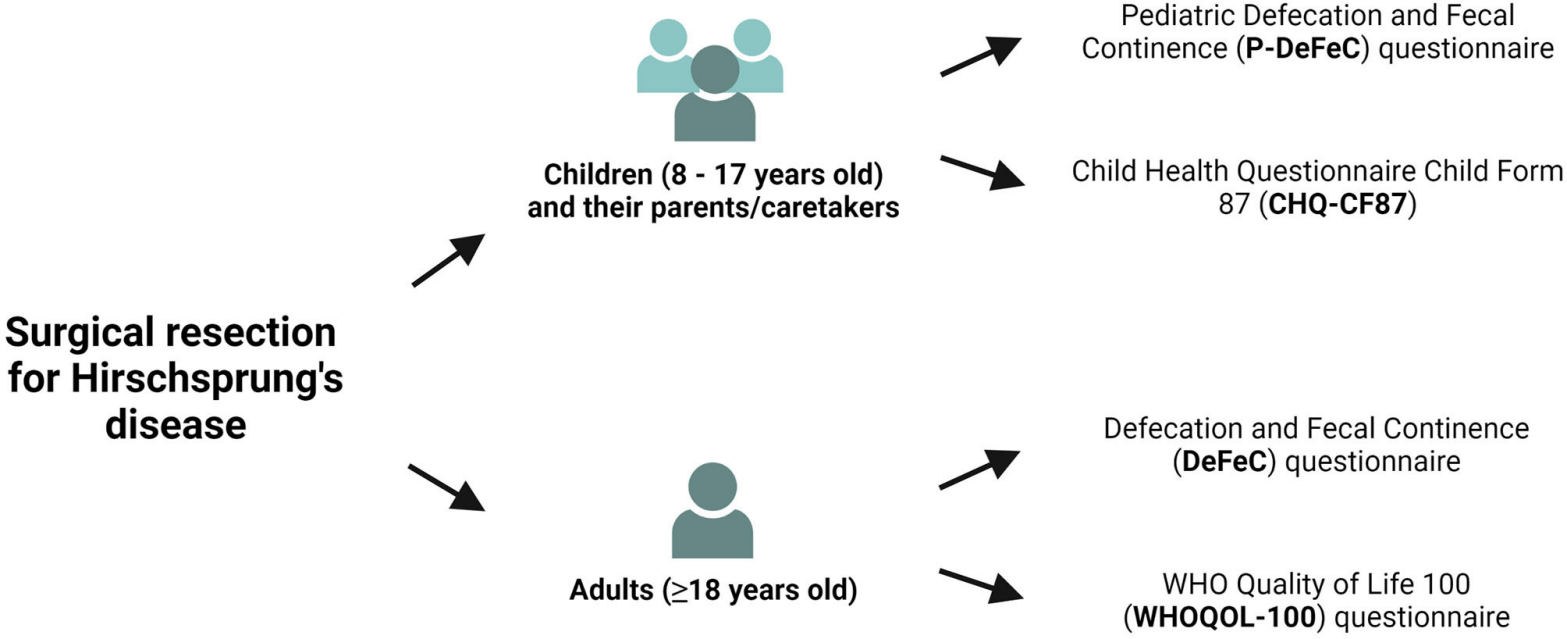
Flowchart of the study design.

We sent the under 18-year-old patients the Pediatric Defecation and Fecal Continence questionnaire (P-DeFeC) (10, 11) as well as the Child Health Questionnaire Child Form 87 (CHQ-CF87, Figure 1) (12). We sent the adult participants the Defecation and Fecal Continence (DeFeC) (10) and the WHO Quality of Life 100 (WHOQOL-100, Figure 1) questionnaires (13). All four questionnaires were validated for use in the Netherlands (10, 11, 14, 15).

Although the questions of the P-DeFeC questionnaire are adapted to the level of children, its contents is analogous to the questions of the adult DeFeC questionnaire. Both questionnaires incorporate various validated scoring systems of bowel and urinary function as well as demographic information, medical history, and familial occurrence of Hirschsprung's disease. Familial Hirschsprung's disease was defined as a patient's self-report on the disease in first- (the patient's parents and/or children), second- (the patient's grandparents and/or siblings), or third- or fourth-degree relatives (the patient's uncles and aunts and/or niblings and/or cousins).

### Bowel Function

We assessed the prevalence of constipation in accordance with the Rome IV criteria. Patients are defined as constipated if they suffered from at least two of the six symptoms described here: more than 25% straining when defecating, sensation of incomplete defecation, sensation of anal blockage, hard/lumpy stool, a defecation frequency of less than three times per week, using manual maneuvers when defecating. In addition, also part of the Rome IV criteria, is that constipated patients are rarely able to defecate without laxatives (16). The Rome IV criteria for assessing constipation in children also include a rectal examination by a clinician. However, given the design of this study this was not possible. We therefore applied the adult Rome IV criteria instead. We also applied the Rome IV criteria to determine the prevalence of fecal incontinence (17). Fecal incontinence is defined as suffering from at least two to four incidents of involuntary loss of liquid or solid stool during a 4-week period in the past 6 months. To analyze the severity of constipation we used the validated Agachan score, which ranges from 0 to 30 (18). To analyze the severity of fecal incontinence we used the incontinence score developed by Jorge and Wexner, which ranges from 0 to 20 (19). The higher the score, the more severe the constipation or the fecal incontinence. The use of laxatives, enemas, antidiarrheals, and/or colonic irrigations was defined as usage of the treatment during the past 6 months for at least several times a month.

### Generic Quality of Life in Children

To assess the generic quality of life in children we used the CHQ-CF87 questionnaire (12). It is divided into 10 domains and two single-item questions. The domain scores range from 0 to 100, with 100 indicating the highest quality of life. For this study we focused on the eight domains that are related most to functioning in daily life, including physical functioning, mental health, and self-esteem.

### Generic Quality of Life in Adults

To assess the generic quality of life in adults we used the WHOQOL-100 questionnaire (13). It consists of multiple facets that form six domains, for example, physical, psychological, and social health domains and overall quality of life. The scores in every domain or facet range between 4 and 20 points, where a higher score equals better quality of life. For this questionnaire, all six domains were analyzed, together with selected facets that provided the best impression of functioning in daily life, for example, energy, concentration and work capacity.

### Statistical Analysis

In case of a normal distribution, we present the results as means and standard deviations. For the variables with a skewed distribution, we report medians and interquartile ranges. For the quality of life data we used means and standard deviations for every analysis, because these were Likert scales. We used numbers and percentages to present categorical variables. With regard to comparisons, we used the chi-square test for categorical variables and Student's *T*-test or Mann–Whitney test for continuous variables. We considered *p*-values of less than 0.05 as statistically significant. We performed univariable and multivariable binary logistic regression analyses to determine the likelihood of constipation and fecal incontinence and to adjust for theoretical confounding variables. Potential interactions were checked. The software we used for the statistical analysis was IBM SPSS Statistics, Version 23.0 (Armonk, NY, USA: IBM Corp.). We created the figures with Graphpad Prism, Version 9.1.0 (GraphPad Software, California, USA) and BioRender.com.

### Medical Ethical Review

A local certified Medical Ethical Review Board approved this study (approval code METc 2013/226, University Medical Center Groningen, the Netherlands).

## Results

### Patient Characteristics

**Figure 2:**
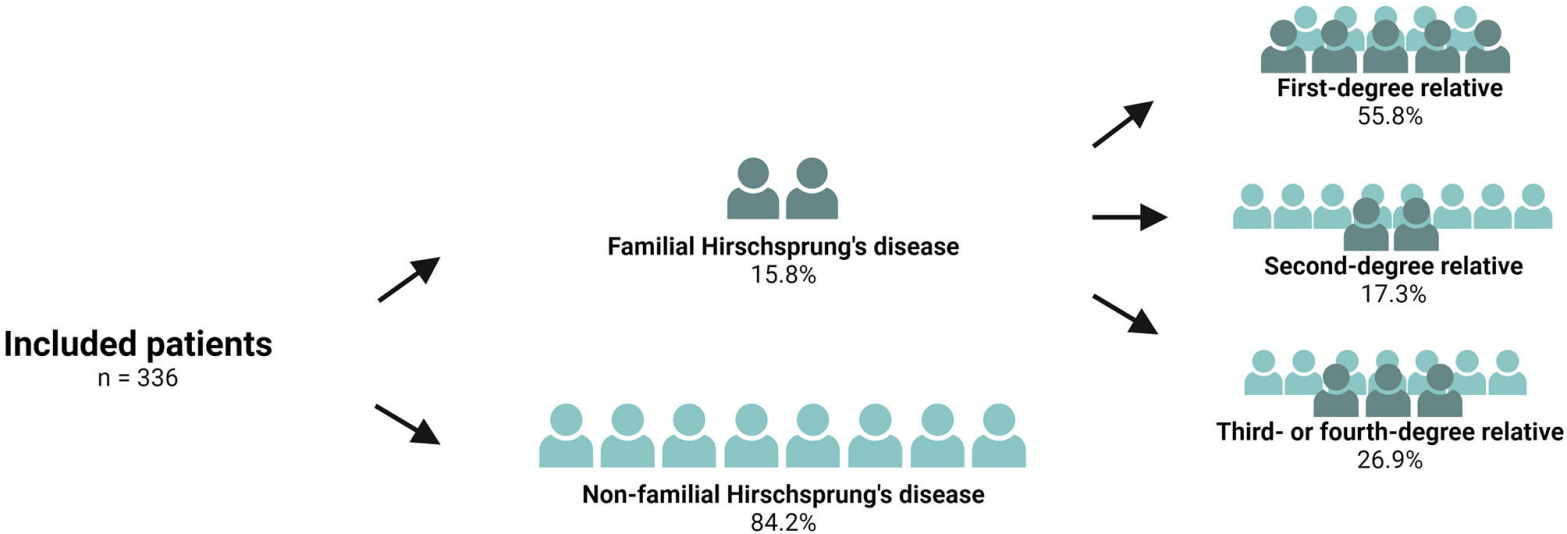
Flowchart of the included patients with familial and non-familial Hirschsprung's disease.

The study comprised a total of 830 patients who had undergone surgery for Hirschsprung's disease. We excluded 57 patients with an unknown or foreign address, 25 patients with a permanent stoma, 86 patients who were intellectually disabled, and 43 deceased patients. Of the remaining 619 patients, 346 patients returned both questionnaires, representing a response rate of 55.9%. A comparison of the responders vs. the non-responders revealed that the responders were younger, as has been described previously (8). We excluded ten patients with an ultrashort aganglionic segment. Finally, the study population comprised 336 patients, 53 (15.8%) of whom were patients with familial Hirschsprung's disease and 283 (84.2%) were non-familial Hirschsprung patients (Figure 2). Of the 53 patients with familial Hirschsprung's disease, the closest family member with the same disease was a first-degree relative in 55.8% of the cases, a second-degree relative in 17.3%, and a third- or fourth-degree relative in 26.9% of the cases (Figure 2).

We present a comparison of demographic and clinical characteristics between familial and non-familial Hirschsprung patients in Table 1. The prevalence of long-segment or total colonic aganglionosis was significantly higher in the patients with familial Hirschsprung's disease than in the non-familial patients (17.0 and 13.2% vs. 7.1 and 6.4%, respectively, *p* = 0.009).

**Table 1:** Characteristics of patients with familial vs. non-familial Hirschsprung's disease.

	**Familial no. (%)**	**Non-familial no. (%)**	***p-*value**
Overall	53 (100.0)	283 (100.0)	
Male patients	42 (79.2)	224 (79.2)	0.988
Age at follow-up (years)^a^	19.0 (8.0–42.0)	17.0 (8.0–45.0)	0.270
Age at surgery (months)^a^	6.3 (0.5–160.4)	5.9 (0.3–169.4)	0.753
Length of aganglionosis			
Rectosigmoid	37 (69.8)	245 (86.6)	0.009^*^
Long-segment	9 (17.0)	20 (7.1)	
Total colonic	7 (13.2)	18 (6.4)	
Congenital comorbidities	5 (9.4)	26 (9.2)	0.955
Preoperative enterocolitis	6 (11.3)	38 (13.4)	0.676
Preoperative stoma	29 (54.7)	137 (48.4)	0.399
Type of reconstruction			
Duhamel	29 (56.9)	178 (63.1)	0.171
Soave	1 (2.0)	0 (0.0)	
Rehbein	13 (25.5)	56 (20.9)	
Swenson	0 (0.0)	1 (0.4)	
Transanal pull-through	8 (15.7)	44 (15.6)	
Surgical approach			
Laparotomic	35 (68.6)	207 (73.4)	0.622
Laparoscopic	8 (15.7)	31 (11.0)	
Combined transanal	8 (15.7)	44 (15.6)	
Anal sphincterotomy	6 (11.3)	11 (3.9)	0.023*
Anal dilatation	12 (22.6)	61 (21.6)	0.860
Postoperative complication	3 (5.8)	31 (11.0)	0.252
Postoperative enterocolitis	12 (22.6)	36 (12.7)	0.058
Redo pull-through	2 (3.8)	21 (7.4)	0.335

a *Values are expressed as medians ± ranges*.

* *Statistical significance of p < 0.05*.

### Likelihood and Treatment of Constipation

Univariable regression analysis did not show a significantly different probability of constipation in patients with or without familial Hirschsprung's disease (Table 2). However, when we adjusted for length of aganglionosis, sex, and age at follow-up in multivariable regression analysis, we found that patients with familial Hirschsprung's disease were more than twice as likely to suffer from constipation (OR 2.47, 95% CI, 1.21–5.05, *p* = 0.013, Table 2). Patients with a second- to fourth-degree relative with Hirschsprung's disease had an increased likelihood of constipation, compared to patients with a first-degree relative, following univariable regression analysis (OR 3.69, 95% CI, 1.04–13.12, *p* = 0.043). The difference was no longer significant in multivariable regression analysis.

**Table 2:** The likelihood of constipation and fecal incontinence.

	**Univariable logistic regression**		**Multivariable logistic regression**	
	**Odds ratio (95% CI)**	***p*-value**	**Odds ratio (95% CI)**	***p*-value**
**Constipation** ^**a**^				
Familial Hirschsprung's disease				
No	Reference		Reference	
Yes	1.79 (0.93–3.46)	0.081	2.47 (1.21–5.05)	0.013^*^
Degrees of consanguinity with				
relatives with Hirschsprung's disease				
First degree	Reference		Reference	
Second to fourth degree	3.69 (1.04–13.12)	0.043^*^	2.22 (0.55–8.98)	0.262
**Fecal incontinence** ^**b**^				
Familial Hirschsprung's disease				
No	Reference		Reference	
Yes	0.84 (0.43–1.65)	0.612	0.92 (0.45–1.86)	0.809
Degrees of consanguinity with				
relatives with Hirschsprung's disease				
First degree	Reference		Reference	
Second to fourth degree	1.11 (0.31–3.91)	0.872	1.72 (0.35–8.50)	0.505

a *Multivariable analysis was adjusted for length of the aganglionosis, sex, and age at follow-up*.

b *Multivariable analysis was adjusted for length of the aganglionosis, redo pull-through, and age at follow-up*.

*CI, confidence interval*.

* *Statistical significance of p < 0.05*.

**Figure 3:**
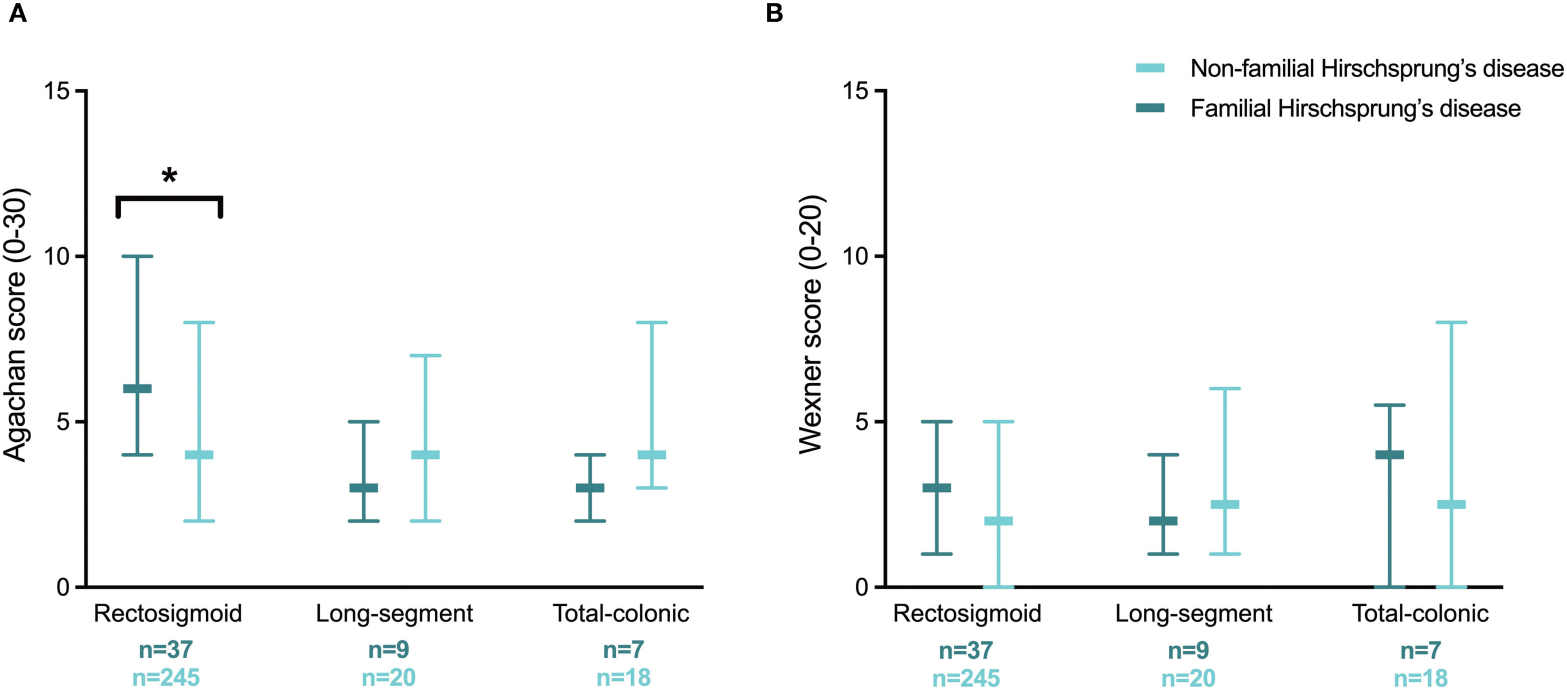
Severity of constipation **(A)** and fecal incontinence **(B)** in patients with familial and non-familial Hirschsprung's disease for different categories of aganglionic length. Values are reported as medians with interquartile ranges. *Statistical significance of *p* < 0.05.

We also compared the severity of constipation in the three categories of aganglionic length between patients with and without familial Hirschsprung's disease (Figure 3A). A significantly worse median Agachan score was found in patients with familial Hirschsprung's disease in the rectosigmoid category (4.0 vs. 6.0, *p* = 0.024). The Agachan score was not significantly different between patients with a first-degree relative with Hirschsprung's disease vs. a second- to fourth-degree relative (median 5.0 vs. 6.0, respectively, *p* = 0.255).

**Figure 4:**
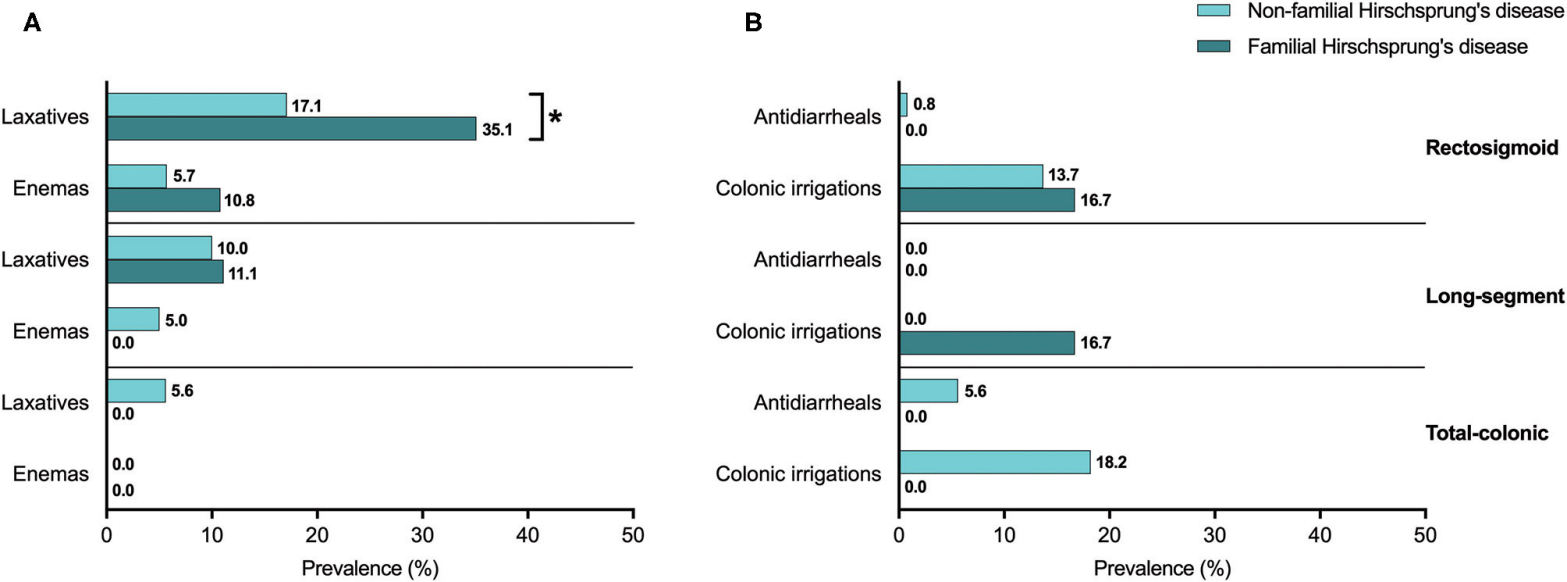
**(A,B)** The use of defecation treatment in patients with familial and non-familial Hirschsprung's disease. *Statistical significance of *p* < 0.05.

When we looked at treatments used for constipation, patients with familial Hirschsprung's disease of the rectosigmoid used laxatives twice as often as patients without familial Hirschsprung's disease of the rectosigmoid (35.1 vs. 17.1% respectively, *p* = 0.010, Figure 4A). For patients with long-segment or total colonic aganglionosis we found no difference in the use of constipation treatments between patients with and without familial Hirschsprung's disease (Figure 4A).

### Likelihood and Treatment of Fecal Incontinence

In contrast to constipation, both univariable and multivariable regression analysis showed no significantly different likelihood of fecal incontinence between patients with familial vs. non-familial Hirschsprung's disease (Table 2). Likewise, the association between fecal incontinence and a first-degree relative with Hirschsprung's disease vs. a second- to fourth-degree relative was also not significantly different. The severity of fecal incontinence was comparable for patients with and without familial Hirschsprung's disease within the three categories of aganglionic length (Figure 3B). The Wexner score was also not significantly different between patients with a first-degree relative with Hirschsprung's disease vs. a second- to fourth-degree relative (median 3.0 vs. 2.0, respectively, *p* = 0.787).

The prevalence of treatments related to fecal incontinence, including the use of colonic irrigations or antidiarrheals, was not significantly different between patients with and without familial Hirschsprung's disease of the rectosigmoid, long-segment, or total colon (Figure 4B).

### Generic Quality of Life in Children

We determined the generic quality of life in 146 pediatric patients, among whom there were 19 (13.0%) children with familial Hirschsprung's disease. We found no significant difference for any of the investigated domain scores of the CHQ-CF87 between patients with and without familial Hirschsprung's disease of the rectosigmoid (Table 3). The number of patients with familial long-segment or total colonic aganglionosis was exceedingly small (*n* = 3 and *n* = 2, respectively). The only significant different score was in the domain “bodily pain” among familial vs. non-familial patients with total colonic aganglionosis (40.0 vs. 76.4, *p* = 0.046, Supplementary Table 1). Children with a first-degree relative vs. a second- to fourth-degree relative with Hirschsprung's disease showed a significantly higher score for “general behavior” (84.4 vs. 63.3, *p* = 0.021, Supplementary Table 2).

**Table 3:** Generic quality of life in pediatric patients with Hirschsprung's disease of the rectosigmoid.

**Domains of the** **CHQ-CF87**	**Familial** **(*n* = 14)**	**Non-familial** **(*n* = 110)**	***p-*value**
	**Mean (SD)**	**Mean (SD)**	
Physical functioning	97.9 (4.8)	97.6 (7.9)	0.899
Bodily pain	82.9 (21.6)	80.4 (22.1)	0.691
General behavior	73.6 (20.8)	71.9 (20.5)	0.776
Mental health	77.0 (12.2)	78.3 (13.1)	0.723
Self-esteem	73.2 (13.4)	76.3 (13.5)	0.420
General health perceptions	75.1 (17.2)	73.7 (19.5)	0.794
Family activities	90.2 (16.8)	86.1 (17.9)	0.415
Family cohesion	71.4 (18.4)	74.8 (20.8)	0.567

*CHQ-CF, Child Health Questionnaire Child Form*.

### Generic Quality of Life in Adults

In the same way, we analyzed the 155 adult patients, 26 (16.8%) of whom had familial Hirschsprung's disease. The domain scores on the WHOQOL-100 were all comparable between patients with and without familial Hirschsprung's disease of the rectosigmoid (Table 4). Looking at the facets, significantly higher scores for “energy/fatigue” (16.3 vs. 14.4, respectively, *p* = 0.029), “thinking/learning/concentration” (16.5 vs. 15.5, respectively, *p* = 0.024), and “work capacity” (18.3 vs. 16.9, respectively, *p* = 0.036) were found in the familial patients vs. the non-familial patients with Hirschsprung's disease of the rectosigmoid. Once again the number of patients with familial long-segment or total colonic aganglionosis was exceedingly small (*n* = 3 and *n* = 5, respectively). No significant different domain or facet scores were found between these familial patients vs. the non-familial patients (Supplementary Table 3). Adults with a first-degree relative vs. a second- to fourth-degree relative with Hirschsprung's disease showed a significantly lower score for “thinking/learning/concentration” (15.6 vs. 17.2, *p* = 0.033, Supplementary Table 4).

**Table 4:** Generic quality of life in adult patients with Hirschsprung's disease of the rectosigmoid.

**Domains/Facets of the WHOQOL-100**	**Familial (*n* = 18)**	**Non-familial (*n* = 110)**	***p-*value**
	**Mean (SD)**	**Mean (SD)**	
**Physical Health**	16.8 (2.9)	15.5 (2.7)	0.053
Energy and fatigue	16.3 (3.5)	14.4 (3.4)	0.029^*^
**Psychological**	16.4 (2.0)	15.5 (2.3)	0.121
Thinking, learning, and concentration	16.5 (1.5)	15.5 (2.6)	0.024^*^
Self-esteem	16.4 (2.1)	15.2 (2.7)	0.061
**Independence level**	18.3 (1.8)	17.5 (2.3)	0.157
Work capacity	18.3 (2.2)	16.9 (2.8)	0.036^*^
**Social relations**	16.2 (2.2)	15.8 (2.6)	0.474
Personal relationships	17.3 (2.0)	16.4 (2.7)	0.189
**Environment**	16.7 (1.4)	16.2 (2.1)	0.277
**Spirituality/religion/personal beliefs**	13.3 (4.4)	12.9 (4.4)	0.681
**Quality of life from the point of view of the evaluated subject**	17.1 (2.3)	16.2 (2.8)	0.208

*WHOQOL, WHO Quality of Life*.

* *Statistical significance of p < 0.05*.

## Discussion

Despite worse bowel function problems, patients with familial Hirschsprung's disease of the rectosigmoid show better physical and psychosocial generic quality of life upon reaching adulthood.

In our study population 15.8% of the patients reported having a relative who had also been born with Hirschsprung's disease. This percentage is comparable to the average prevalence of 20% found in the literature (2–4). It is known that longer aganglionic lengths are associated with a higher familial occurrence (2–4) and this fact corresponds with our findings. We found that patients with familial Hirschsprung's disease were twice more likely to suffer from constipation when stratified for length of aganglionosis, sex, and age. These are three well-known confounders (16), although the reported effect of aging on constipation in Hirschsprung patients varies widely between relief (20, 21) and persistence (21–23). Unfortunately, our data did not enable us to indicate the cause of the increased likelihood of constipation in patients with familial Hirschsprung's disease. Nevertheless, awareness should be raised for the possibility of more severe constipation in children with familial Hirschsprung's disease of the rectosigmoid. The fact that laxatives were used by twice as many patients with familial Hirschsprung of the rectosigmoid may either be caused by more severe disease or by more willingness to take laxatives because their environment may be more familiar with the treatment of Hirschsprung's disease.

Despite the increased likelihood of constipation in patients with familial Hirschsprung's disease, the overall and physical quality of life was comparable between the familial and non-familial patients in children and adults alike. This may be explained by the fact that not constipation but fecal incontinence is considered the main cause of impaired physical quality of life in Hirschsprung patients (7, 23–26), which was not found to be increased in familial cases. Apart from physical quality of life, previous studies found impaired psychosocial quality of life in Hirschsprung patients (26, 27). Although we found the psychosocial domains to be comparable between pediatric familial and non-familial patients, energy, concentration, and work capacity of adult patients with familial Hirschsprung's disease was better, despite their worse bowel function. That we found better quality of life in facets indicative of functioning in daily life could possibly derive from the exemplary role of a relative with the same disease. This may not only lead to realistic expectations of long-term outcomes, but also to small adaptations in how to live “a normal life” despite the disease. Previously, it was reported that there is a low ability to recognize Hirschsprung-related symptoms in adult healthcare (28). Patients with familial Hirschsprung may be at an advantage regarding the recognition of bowel function problems and may possibly find it easier to talk about these problems, which might improve their ability to cope. Based on our results, it is likely that families who are familiar with Hirschsprung's disease provide a better environment for developing good coping strategies than families new to the disease.

In addition, the fact that families who are familiar with Hirschsprung's disease experience a lower level of disease-related stress may affect the way patients experience their own condition. This assumption is justified by high levels of stress and anxiety in parents of patients with Hirschsprung's disease, as has been recognized by others (7, 24, 29, 30). Our results emphasize the need for more familial involvement in the long-term follow-up of Hirschsprung's disease, because familial coping seems to have a positive influence on the ability to cope of the patients themselves, as has been suggested before (6, 7).

This is the first study to compare bowel function and generic quality of life in patients with familial and non-familial Hirschsprung's disease. We acknowledge that this was not a longitudinal study, which precludes analysis of how bowel function and quality of life developed over time. Comparison between patients with first- vs. second- to fourth-degree relatives with Hirschsprung's disease did not reveal many differences in either bowel function or quality of life, but the number of patients per subgroup was exceedingly small. Theoretically, a close relative could play a considerable role in the patient's life and may have more influence than if such a relative were absent. Unfortunately, the rarity of Hirschsprung's disease complicates reporting on large groups of patients. For the same reason the comparison of quality of life between familial and non-familial patients with long-segment and total-colonic aganglionosis was limited. Another limitation was the exclusion of patients with a permanent stoma. This could theoretically have biased our results because patients with intractable fecal incontinence may sometimes receive a permanent stoma. However, seeing that only 25 out of 830 patients were excluded because of a permanent stoma, no substantial bias is expected from this lack of data. Finally, data about familial occurrence of Hirschsprung's disease was gathered through self-report by the patients. As a result, we may have missed cases in which patients were unaware of any relatives who also suffered from Hirschsprung's disease. Nevertheless, also in the clinical setting, familial occurrence of the disease is usually assessed by self-report. Besides, if patients are not aware of the familial occurrence of the disease, no exemplary role could stem from the relative or relatives with the same disease either.

## Conclusion

Despite a higher prevalence and severity of constipation, different facets of generic quality of life are better in patients with familial Hirschsprung's disease of the rectosigmoid. Familial experience with the disease seems to have a positive influence on patients' own ability to cope.

## Data Availability Statement

The original contributions presented in the study are included in the article/Supplementary Material, further inquiries can be directed to the corresponding author/s.

## Ethics Statement

The studies involving human participants were reviewed and approved by Medical Ethical Review Board of the University Medical Center Groningen. Written informed consent to participate in this study was provided by the participants' legal guardian/next of kin.

## Author Contributions

SV, RM, MT, and PB conceived the research idea, finalized the methods, and analyzed the data. SV wrote the first draft. SV, RM, AS, CS, EH, IB, WG, MW, KV, MT, and PB contributed to data collection, interpretation of the results, and drafting and finalizing the manuscript. All authors contributed to the article and approved the submitted version.

## Conflict of Interest

The authors declare that the research was conducted in the absence of any commercial or financial relationships that could be construed as a potential conflict of interest.

## Publisher's Note

All claims expressed in this article are solely those of the authors and do not necessarily represent those of their affiliated organizations, or those of the publisher, the editors and the reviewers. Any product that may be evaluated in this article, or claim that may be made by its manufacturer, is not guaranteed or endorsed by the publisher.

## Abbreviations

CHQ-CF: Child Health Questionnaire Child Form
DeFeC: defecation and fecal continence
P-DeFeC: pediatric defecation and fecal continence
WHOQOL-100: World Health Organization Quality of Life Assessment Instrument.
