# Supplementary material for: Familial Experience With Hirschsprung's Disease Improves the Patient's Ability to Cope
Source: Front Pediatr. 2022 Mar 7;10:820976. doi: 10.3389/fped.2022.820976 (PMC8935079; doi:10.3389/fped.2022.820976)
Supplement: Supplementary file 1 [file Table_1.DOCX]

|  | **Long-segment** | | | **Total colonic** | | |
| --- | --- | --- | --- | --- | --- | --- |
| **Domains of the CHQ-CF87** | **Familial**  n = 3  Mean (SD) | **Non-familial**  n = 6  Mean (SD) | ***p* value** | **Familial**  n = 2  Mean (SD) | **Non-familial**  n = 11  Mean (SD) | ***p* value** |
| Physical functioning | 96.3 (3.7) | 99.4 (1.5) | *0.106* | 85.2 (0.0) | 93.3 (11.1) | *0.341* |
| Bodily pain | 66.7 (41.6) | 93.3 (10.3) | *0.159* | 40.0 (0.0) | 76.4 (22.0) | *0.046 ** |
| General behavior | 66.7 (31.8) | 68.3 (12.9) | *0.910* | 72.5 (17.7) | 73.6 (21.3) | *0.945* |
| Mental health | 73.4 (17.2) | 83.3 (6.5) | *0.232* | 71.9 (8.8) | 79.5 (10.7) | *0.366* |
| Self-esteem | 70.2 (19.3) | 81.3 (8.3) | *0.251* | 70.5 (8.8) | 77.6 (6.6) | *0.203* |
| General health perceptions | 60.8 (37.4) | 85.2 (9.4) | *0.153* | 41.3 (19.4) | 65.1 (21.4) | *0.172* |
| Family activities | 88.9 (19.2) | 88.2 (18.0) | *0.959* | 81.3 (2.9) | 81.1 (21.4) | *0.991* |
| Family cohesion | 76.7 (14.4) | 65.8 (24.2) | *0.506* | 72.5 (17.7) | 67.3 (23.1) | *0.769* |

**Supplementary Table 1.** Generic quality of life in pediatric patients with long-segment or total-colonic Hirschsprung’s disease

Abbreviation: CHQ-CF, Child Health Questionnaire Child Form.
