# Supplementary material for: Familial Experience With Hirschsprung's Disease Improves the Patient's Ability to Cope
Source: Front Pediatr. 2022 Mar 7;10:820976. doi: 10.3389/fped.2022.820976 (PMC8935079; doi:10.3389/fped.2022.820976)
Supplement: Supplementary file 2 [file Table_2.DOCX]

**Supplementary Table 2.** Generic quality of life in pediatric patients with first-, or second- to fourth-degree relatives with Hirschsprung’s disease

| **Domains of the CHQ-CF87** | **First-degree**  **relative with Hirschsprung’s disease**  n = 9  Mean (SD) | **Second- to fourth-degree relative with**  **Hirschsprung’s disease**  n = 11  Mean (SD) | ***p* value** |
| --- | --- | --- | --- |
| Physical functioning | 95.5 (6.4) | 97.3 (5.3) | *0.489* |
| Bodily pain | 71.1 (28.0) | 79.1 (25.5) | *0.514* |
| General behavior | 84.4 (15.5) | 63.6 (20.4) | *0.021 ** |
| Mental health | 79.3 (11.8) | 72.9 (11.7) | *0.236* |
| Self-esteem | 77.8 (6.4) | 68.5 (15.6) | *0.114* |
| General health perceptions | 67.7 (22.8) | 72.3 (23.2) | *0.657* |
| Family activities | 95.8 (8.3) | 84.1 (18.3) | *0.094* |
| Family cohesion | 75.6 (15.5) | 72.3 (19.9) | *0.691* |

Abbreviation: CHQ-CF, Child Health Questionnaire Child Form.
