# Supplementary material for: Familial Experience With Hirschsprung's Disease Improves the Patient's Ability to Cope
Source: Front Pediatr. 2022 Mar 7;10:820976. doi: 10.3389/fped.2022.820976 (PMC8935079; doi:10.3389/fped.2022.820976)
Supplement: Supplementary file 3 [file Table_3.DOCX]

|  | **Long-segment** | | | **Total colonic** | | |
| --- | --- | --- | --- | --- | --- | --- |
| **Domains/Facets of the WHOQOL-100** | **Familial**  n = 3  Mean (SD) | **Non-familial**  n = 13  Mean (SD) | ***p* value** | **Familial**  n = 5  Mean (SD) | **Non-familial**  n = 6  Mean (SD) | ***p* value** |
| **Physical Health** | 16.7 (0.9) | 16.1 (2.0) | *0.615* | 14.4 (1.2) | 16.3 (2.2) | *0.123* |
| Energy and fatigue | 14.7 (2.5) | 15.4 (2.9) | *0.703* | 12.0 (1.2) | 14.8 (4.0) | *0.148* |
| **Psychological** | 15.0 (2.0) | 16.6 (1.2) | *0.076* | 15.7 (2.2) | 16.3 (1.1) | *0.585* |
| Thinking, learning, and concentration | 14.7 (3.2) | 16.0 (2.3) | *0.406* | 15.8 (2.3) | 15.8 (1.9) | *0.980* |
| Self-esteem | 14.3 (2.1) | 15.8 (1.1) | *0.095* | 16.0 (1.9) | 16.5 (1.5) | *0.635* |
| **Independence level** | 18.3 (0.3) | 18.0 (1.4) | *0.711* | 17.8 (1.2) | 17.8 (2.1) | *0.970* |
| Work capacity | 17.7 (0.6) | 18.1 (1.8) | *0.508* | 16.4 (3.5) | 17.3 (2.7) | *0.627* |
| **Social relations** | 16.2 (0.7) | 16.9 (1.9) | *0.571* | 15.4 (2.5) | 17.2 (2.6) | *0.281* |
| Personal relationships | 16.3 (0.6) | 17.8 (1.3) | *0.083* | 15.8 (3.4) | 18.2 (1.5) | *0.157* |
| **Environment** | 16.0 (1.0) | 17.1 (1.3) | *0.206* | 16.5 (1.0) | 17.1 (1.3) | *0.407* |
| **Spirituality/religion/personal beliefs** | 12.0 (1.0) | 13.1 (3.1) | *0.570* | 12.0 (4.0) | 10.0 (4.9) | *0.481* |
| **Quality of life from the point of view of the evaluated subject** | 16.7 (1.5) | 17.2 (1.8) | *0.623* | 16.0 (0.0) | 16.7 (1.5) | *0.328* |

**Supplementary Table 3.** Generic quality of life in adult patients with long-segment or total-colonic Hirschsprung’s disease

Abbreviation: WHOQOL, WHO Quality of Life.
