# Supplementary material for: Familial Experience With Hirschsprung's Disease Improves the Patient's Ability to Cope
Source: Front Pediatr. 2022 Mar 7;10:820976. doi: 10.3389/fped.2022.820976 (PMC8935079; doi:10.3389/fped.2022.820976)
Supplement: Supplementary file 4 [file Table_4.DOCX]

**Supplementary Table 4.** Generic quality of life in adult patients with first-, or second- to fourth-degree relatives with Hirschsprung’s disease

| **Domains/Facets of the WHOQOL-100** | **First-degree**  **relative with Hirschsprung’s disease**  n = 17  Mean (SD) | **Second- to fourth-degree relative with**  **Hirschsprung’s disease**  n = 9  Mean (SD) | ***p* value** |
| --- | --- | --- | --- |
| **Physical Health** | 15.9 (2.3) | 17.2 (3.1) | *0.239* |
| Energy and fatigue | 14.7 (3.3) | 16.4 (3.8) | *0.232* |
| **Psychological** | 15.7 (1.8) | 16.8 (2.2) | *0.175* |
| Thinking, learning, and concentration | 15.6 (1.9) | 17.2 (1.5) | *0.033 ** |
| Self-esteem | 15.9 (1.8) | 16.4 (2.7) | *0.572* |
| **Independence level** | 17.9 (1.5) | 18.7 (1.7) | *0.251* |
| Work capacity | 17.6 (2.7) | 18.4 (1.9) | *0.405* |
| **Social relations** | 16.0 (2.0) | 16.1 (2.4) | *0.903* |
| Personal relationships | 16.7 (2.3) | 17.2 (2.2) | *0.585* |
| **Environment** | 16.5 (1.1) | 16.8 (1.5) | *0.557* |
| **Spirituality/religion/personal beliefs** | 13.4 (3.5) | 12.1 (4.9) | *0.463* |
| **Quality of life from the point of view of the evaluated subject** | 16.4 (1.9) | 17.7 (1.9) | *0.129* |

Abbreviation: WHOQOL, WHO Quality of Life.
